# Novel Hole Transporting Materials Based on 4-(*9H*-Carbazol-9-yl)triphenylamine Derivatives for OLEDs

**DOI:** 10.3390/molecules190914247

**Published:** 2014-09-10

**Authors:** Quynh Pham Bao Nguyen, Sueng Ji Baek, Mi Jin Kim, Na Young Shin, Gyeong Woo Kim, Dong Cheol Choe, Jang Hyuk Kwon, Kyu Yun Chai

**Affiliations:** 1 The Division of Bio-Nanochemistry, College of Natural Sciences and the Institute for Basic Science, Wonkwang University, Iksan City, Chonbuk 570-749, Korea; 2 Department of Information Display, Kyung Hee University, Dongdaemoon-gu, Seoul 130-701, Korea; 3 Venture Building, #824, Palbokdong 2-ga, Deokjin-gu, Jeonju, Chonbuk 561-844, Korea

**Keywords:** organic light emitting diodes, hole-transporting material, 4-(9*H*-carbazol-9-yl)triphenylamine, carbazole, triarylamine

## Abstract

During the past few years, organic light emitting diodes (OLEDs) have been increasingly studied due to their emerging applicability. However, some of the properties of existing OLEDs could be improved, such as their overall efficiency and durability; these aspects have been addressed in the current study. A series of novel hole-transporting materials (HTMs) **3a**–**c** based on 4-(9*H*-carbazol-9-yl)triphenylamine conjugated with different carbazole or triphenylamine derivatives have been readily synthesized by Suzuki coupling reactions. The resulting compounds showed good thermal stabilities with high glass transition temperatures between 148 and 165 °C. The introduction of HTMs **3b** and **3c** into the standard devices ITO/HATCN/NPB/HTMs **3** (indium tin oxide/dipyrazino(2,3-f:2',3'-h)quinoxaline 2,3,6,7,10,11-hexacarbonitrile/*N,N'*-bis(naphthalen-1-yl)-*N,N'*-bis(phenyl)-benzidine/HTMs)/CBP (4,4'-Bis(N-carbazolyl)-1,1'-biphenyl): 5% Ir(ppy)_3_/Bphen/LiF/Al (tris[2-phenylpyridinato-C2,N]iridium(III)/4,7-diphenyl-1,10-phenanthroline/LiF/Al) resulted in significantly enhanced current, power, and external quantum efficiencies (EQE) as compared to the reference device without any layers of HTMs **3**.

## 1. Introduction

Organic light emitting diodes (OLEDs) have recently attracted remarkable attention for their applications in full-color flat-panel displays and solid-state lighting. Great progress has been made to improve OLED device performance in order to obtain higher efficiency and better durability [1]. It is well established that one of the main reasons for the degradation of the OLED device is the morphological change in the amorphous organic layers, especially of the hole transport layer, caused by Joule heating during device operation [2]. In order to overcome this issue, it is necessary to develop amorphous materials with a high glass transition temperature (*T_g_*) [3]. Therefore, different synthetic approaches have been developed to produce novel high *T_g_* hole-transport materials (HTMs) to generate thermally stable OLEDs [4,5,6,7].

**Figure 1:**
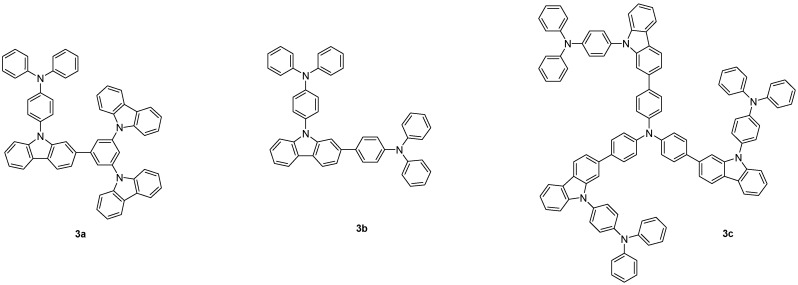
Structures of the designed HTMs **3**.

Triphenylamine and carbazole, with their strong electron donating nature, have been reported to exhibit excellent hole-transport properties and to play an important part in the development of HTMs [2,3,4,5,6,7,8,9,10,11,12,13,14,15,16,17,18]. Therefore, the incorporation of these two moieties in the same molecule is a very attractive means to achieve both good thermal stability and hole-transport characteristics. The weak thermal stability of triphenylamine could be greatly improved upon incorporation of the rigid and highly stable carbazole moiety [5] which, however, is a weaker electron donor than the diphenylamine moiety [18]. In this paper, we wish to report a series of novel HTMs based on 4-(9*H*-carbazol-9-yl)triphenylamine. In view of effective molecular design aspects, different carbazole or triphenylamine derivatives were conjugated at the carbazole ring of 4-(9*H*-carbazol-9-yl)triphenylamine to improve the thermal stability, the morphological stability, and the hole-transport property (Figure 1). With these molecular architectures, amorphous HTMs with high *T_g_* were obtained. The results of the synthesis, characterization, and fabrication of OLED devices based on the novel HTMs have also been communicated in the present study.

## 2. Results and Discussion

**Scheme 1:**
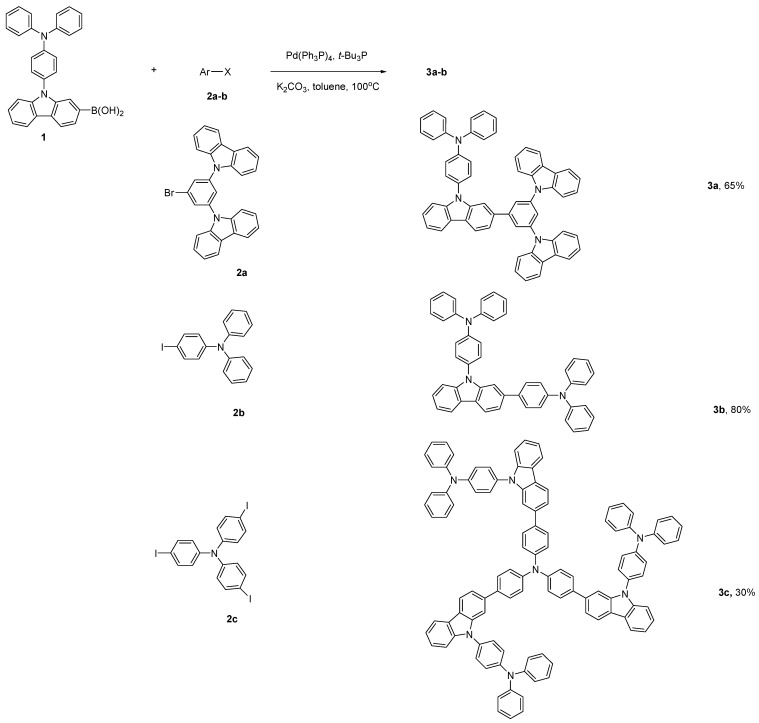
Synthesis of HTMs **3**.

The designed HTMs **3a**–**c** based on 4-(9*H*-carbazol-9-yl)triphenylamine derivatives were readily prepared by Suzuki coupling reactions between the commercially available 9-(4-(diphenylamino)phenyl)-9*H*-carbazol-2-yl-2-boronic acid (**1**) and three different aryl halide derivatives, namely 9-(3-bromo-5-(9*H*-carbazol-9-yl)phenyl)-9*H*-carbazole (**2a**), *N*-(4-iodophenyl)-*N*-phenylbenzenamine (**2b**) and tris(4-iodophenyl)amine (**2c**) (Scheme 1). As a result, compounds **3a** and **3b** were obtained in good yields of 65% and 80%, respectively; however, compound **3c** was synthesized in a low yield of 30% due to the three reaction sites and steric hindrances. All compounds **3a**–**c** were highly soluble in dichloromethane or chloroform at room temperature. Consequently, a uniform, stable, and non-crystalline thin film could be obtained from solution casting.

Prior to the fabrication of OLED devices, the novel HTMs **3a**–**c** were identified and characterized by NMR, IR, GC-Mass, thermal decomposition temperature (*T_d_*), *T_g_*, UV-Vis, cyclic voltammetry (CV), PL (photoluminescence) spectroscopies and highest occupied molecular orbital/lowest unoccupied molecular orbital (HOMO-LUMO) energy levels (Table 1). First, the morphological and thermal properties of **3a**–**c** were investigated by differential scanning calorimetry (DSC) and thermogravimetric analysis (TGA). As expected, the *T_g_* values of all HTMs **3a**–**c**, *i.e.*, 152, 148, and 165 °C, respectively, were significantly higher than the *T_g_* of 100 °C of the widely used HTM *N,N'*-bis(naphthalen-1-yl)-*N,N'*-bis(phenyl)benzidine (NPB) [15]. In addition, HTMs **3a**–**c** showed good thermal stabilities as TGA measurements revealed no decomposition below 456, 300, and 280 °C, respectively. These results indicated that the morphological and thermal stabilities could indeed be significantly improved by the introduction of the extended conjugation of structurally rigid and bulky moieties, especially those of the carbazole derivatives. The improved stability allowed high-quality amorphous thin films to be formed through thermal evaporation and also reduced the device damage. Second, the optical properties of HTMs **3a**–**c** were determined by UV-Vis and PL spectrometries and the results are summarized in Table 1. The absorption spectra of **3a**–**c** were in the near-ultraviolet region (291–313 nm), meaning that they hardly absorbed light at wavelengths longer than 400 nm, implying their transparency to visible light ensuring a high light collecting efficiency. All compounds **3a**–**c** were moderately fluorescent with emission peaks observed in the violet-blue region at 422–469 nm. Third, the electrochemical properties of HTMs **3a**–**c** were examined by CV measurements (Table 1). The HOMO-LUMO energy levels of **3a**–**c** were 5.80/2.26, 6.15/2.89, and 5.78/2.43 eV, respectively and their calculated HOMO-LUMO energy gaps (E_g_) were 3.54, 3.26, and 3.35 eV, respectively. 

**Table 1:** Thermal and photophysical properties of HTMs **3**.

Compound	*T_d_* (°C)	*T_g_* (°C)	UV λ_abs_ (nm)	PL λ_max_ (nm)	HOMO (eV)	LUMO (eV)	T_1_ (eV)	E_g_ (eV)
3a	456	152	291, 310, 338	469	5.80	2.26	2.67	3.54
3b	360	148	313, 343	422	6.15	2.89	2.47	3.26
3c	300	165	309, 337	432	5.78	2.43	2.66	3.35

**Figure 2:**
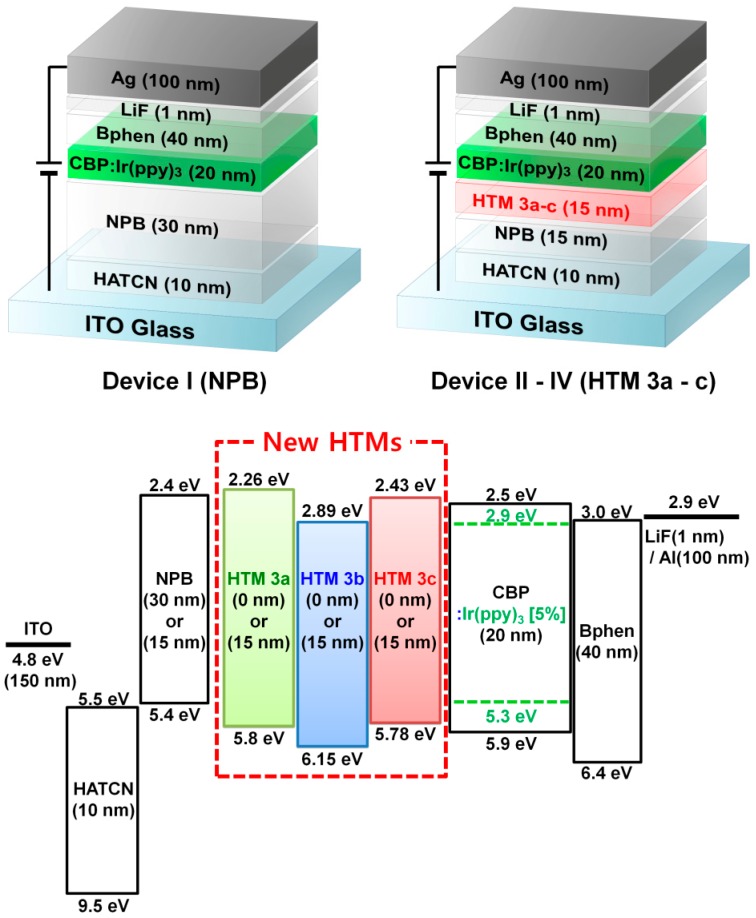
Device structures of designed HTMs **3** and their energy diagrams.

Finally, given the basic advantages of HTMs **3a**–**c**, such as good thermal and morphological stabilities, good photophysical properties as well as proper energy levels, we investigated their hole-transporting performances in green phosphorescent OLEDs with double HTLs (hole transport layers) of NPB/HTMs **3a**–**c** (devices II-IV). In addition, a reference device with a similar structure but without HTMs **3** (device I) was also fabricated to confirm the exciton-blocking as well as the hole-transporting performances of HTMs **3a**–**c** (Figure 2).

**Figure 3:**
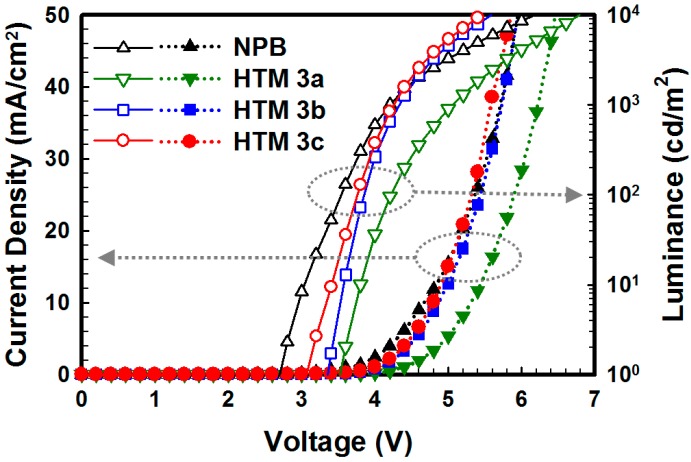
*J-V-L* characteristics of fabricated OLEDs.

The current density-voltage-luminance (*J*-*V*-*L*) characteristics are displayed in Figure 3. HTMs **3b** and **3c** demonstrated very good *J-V-L* performances comparable with NPB, while HTM **3a** showed very poor *J-V-L* characteristics. This could be explained by the fact that the carbazole moiety was a weaker electron donor than the diphenylamine moieties [18]. In addition, HTM **3c** exhibited a slightly better performance than HTM **3b** due to its relatively lower hole injection barrier with NPB.

**Figure 4:**
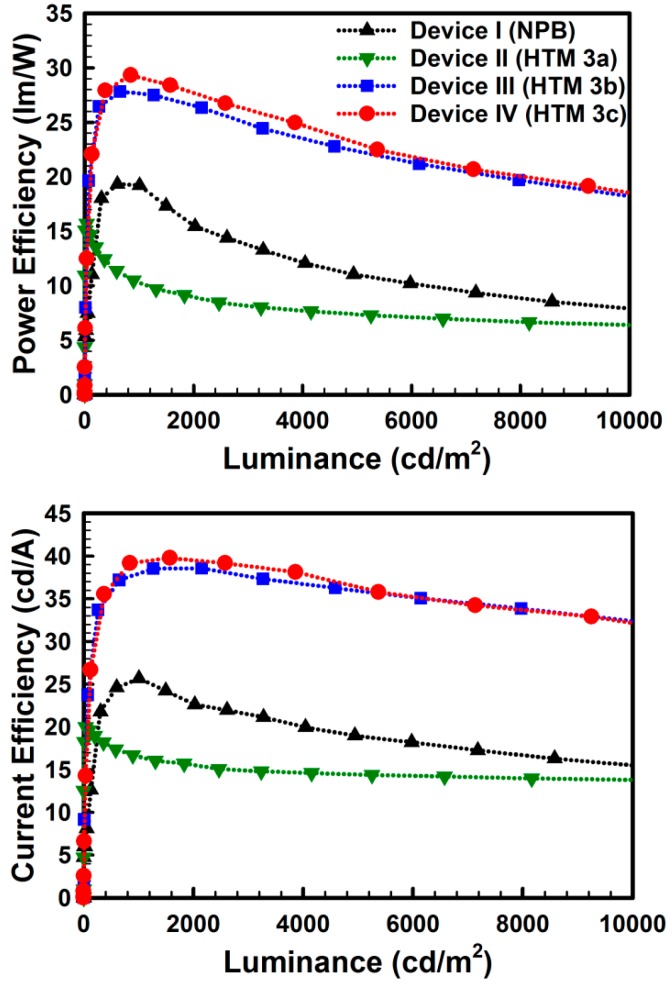
Power & current efficiencies of the fabricated OLEDs.

As seen in Figure 2, in the case of device I, the holes were directly injected into the HOMO of Ir(ppy)_3_ through the HOMO of NPB, which made a narrow recombination zone in EML and led to a lot of singlet-polaron quenching resulting in the low efficiency of device I [19]. On the other hand, the holes in devices III and IV were readily injected into the HOMO of CBP through the HOMO of HTM **3**. The hole flows in the EMLs of devices III and IV were better than that of device I due to the higher hole mobility of the HOMO of CBP than that of the HOMO of Ir(ppy)_3_ in the CBP:Ir(ppy)_3_ layer [20]. Therefore, the charge recombination zones of the devices III and IV were broadened which reduced the singlet-polaron quenching and improved the charge balance in EMLs. For this reason, devices III and IV exhibited high efficiencies (Figure 4). Furthermore, the high triplet energy of HTM **3c** (2.66 eV) also helped improve the efficiency of the device by the triplet exciton confinement in the emissive layer (EML) (Figure 4) [21]. Device IV with HTM **3c** exhibited the highest power and current efficiencies of 29.3 lm/W and 39.8 cd/A, respectively (Table 2).

**Table 2:** Device characteristics.

Characteristics	Device I (NPB)	Device II (HTM 3a)	Device III (HTM 3b)	Device IV (HTM 3c)
Turn-on voltage (1 cd/m^2^)	2.5	3.5	3.4	3.1
Operating voltage (1000 cd/m^2^)	4.2	5.0	4.3	4.2
Efficiency (1000 cd/m^2^)	25.7 cd/A	16.7 cd/A	38.5 cd/A	39.2 cd/A
	19.2 lm/W	10.5 lm/W	27.5 lm/W	29.3 lm/W
Efficiency (Maximum)	25.7 cd/A	20.0 cd/A	38.6 cd/A	39.8 cd/A
	19.3 lm/W	15.7 lm/W	27.8 lm/W	29.3 lm/W
EQE (Maximum)	9.4%	7.0%	13.8%	14.0%
CIE (x, y) (1000 cd/m^2^)	0.31, 0.61	0.29, 0.53	0.31, 0.61	0.31, 0.61

**Figure 5:**
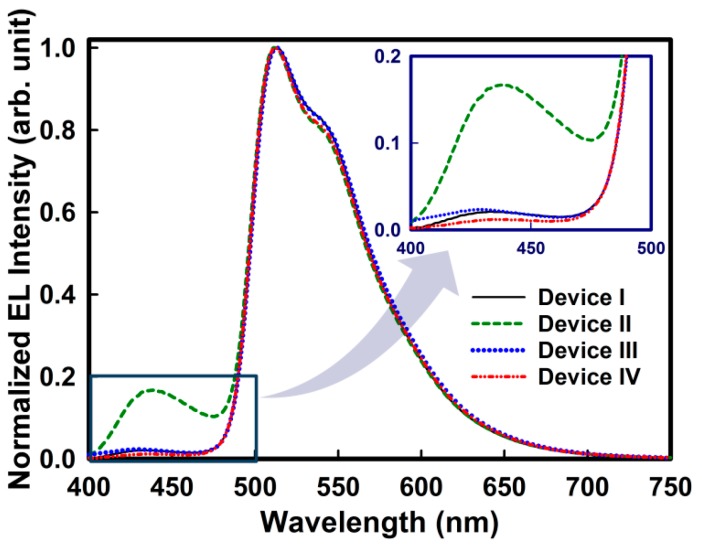
Normalized EL spectra of the fabricated OLEDs at 1000 cd/m^2^. The inset figure shows the enlarged EL spectra in the range of 400 to 500 nm of wavelength.

Figure 5 shows the normalized electroluminescence (EL) spectra of the devices at 1000 cd/m^2^. All the EL spectra showed a maximum peak at 512 nm originating from Ir(ppy)_3_. However, device II exhibited a strong additional peak at 439 nm, generated from the emission of HTM **3a**. The other devices also showed a very weak additional emission peak between 427 and 449 nm (Figure 5), in which device IV exhibited the weakest emission intensity. This meant that the injected electron and hole charges in the EML of the device containing HTM **3c** were well balanced. From all the results above, HTM **3c** seemed the best candidate for an OLED in terms of thermal stability, suitable HOMO level, high triplet energy and device performance. Device IV with HTM **3c** exhibited good overall performance with a low turn on voltage of 3.1 V, a 4.2 V driving voltage at 1000 cd/m^2^, and current and power efficiencies of 39.2 cd/A and 29.3 lm/W, respectively (Table 2).

## 3. Experimental

### 3.1. General Procedures

All reagents and solvents were obtained from commercial suppliers (Aldrich and TCI Chem. Co., Seoul, Korea) and used without further purification. ^1^H- and ^13^C-NMR spectra were recorded on a JEOL JNM-ECP FT-NMR spectrometer operating at 500 and 125 MHz, respectively. IR spectra were obtained on a Shimadzu Prestige-21 FT-IR spectrophotometer. The samples were prepared as a KBr pellet and scanned against a blank KBr pellet background at wave numbers ranging from 4000 to 400 cm^−1^. UV-Vis absorption spectra were recorded on a Scinco S-3100 spectrophotometer while PL spectra were obtained on a CARY Eclipse Varian fluorescence spectrophotometer. The HOMO levels were calculated from oxidation potentials, while the LUMO levels were calculated based on the HOMO levels and the lowest-energy absorption edges of the UV-vis absorption spectra. TGA experiments were conducted on a TG 209F1 (NET-ZSCH) thermal analysis system under a heating rate of 20 °C/min.

### 3.2. Synthesis

#### 3.2.1. Typical Procedure for the Synthesis of Compounds **3a**–**b**

A mixture of 9-(4-(diphenylamino)phenyl)-9*H*-carbazol-2-yl-2-boronic acid (**1**, 2.8 g, 6 mmol), aryl halide **2** (1.5 g, 4 mmol), Pd(Pd_3_P)_4_ (0.47 g, 0.093 mmol), and K_2_CO_3_ (2.8 g, 20 mmol) in toluene (50 mL) and distilled water (25 mL) was stirred overnight at 100 °C under argon. After the completion of the reaction, the mixture was extracted with dichloromethane. The organic layers were separated, dried over magnesium sulfate, filtered, and concentrated. The residues were purified by silica gel column chromatography using *n*-hexane/dichloromethane as the eluent to give the requisite products **3**.

*{4-[2-(3,5-Bis-carbazol-9-yl-phenyl)-carbazol-9-yl]-phenyl}-diphenylamine* (**3a**). Yield: 65%; white solid; FT-IR (KBr pellet): ʋ_max_ 3031, 2923, 1625, 1509, 1235, 1015, 1075 cm^−1^; ^1^H-NMR (CDCl_3_) δ 7.10–8.35 (m, 40H); ^13^C-NMR (CDCl_3_) δ 147.5, 146.0, 142.0, 141.8, 140.7, 139.8, 137.5, 124.1, 123.8, 123.7, 123.6, 123.5, 123.2, 122.9, 122.5, 120.7, 120.6, 120.3, 120.2, 120.1, 119.5, 110.2, 110.0, 109.1, 108.7; GC-MS: 817.13 for C_60_H_41_N_4_ [M+H^+^].

*N-(4-(2-(4-(Diphenylamino)phenyl)-9H-carbazol-9-yl)phenyl)-N-phenylbenzenamine* (**3b**). Yield: 80%; white solid; FT-IR (KBr pellet): ʋ_max_ 2359, 2340, 1509, 1490, 1274, 694 cm^−1^; ^1^H-NMR (CDCl_3_) δ 8.15 (dd, *J* = 7.8 Hz, 2H), 7.59 (s, 1H), 7.00–7.58 (m, 32H); ^13^C-NMR (CDCl_3_) δ 147.8, 147.6, 147.3, 147.1, 141.8,141.7, 139.0, 136.2, 131.3, 129.6, 129.4, 128.3, 128.0, 125.9, 125.0, 124.5, 124.1, 123.9, 123.6, 123.1, 123.0, 122.2, 120.6, 120.3, 120.0, 119.3, 110.0, 107.9; Anal. Calcd for C_48_H_35_N_3_: C, 88.18; H, 5.40; N, 6.43. Found: C, 88.15; H, 5.63; N, 6.50.

#### 3.2.2. Procedure for the Synthesis of Compound **3c**

A mixture of 9-(4-(diphenylamino)phenyl)-9*H*-carbazol-2-yl-2-boronic acid (**1**, 10 g, 22 mmol), tris(4-iodophenyl)amine (**2c**, 3 g, 4.8 mmol), Pd(Pd_3_P)_4_ (1.7 g, 1.47 mmol), *t*-Bu_3_P 50% in toluene (1.4 mL, 2.88 mmol), and K_2_CO_3_ (13 g, 94 mmol) in toluene (150 mL) and distilled water (50 mL) was stirred at 100 °C for 2 days under argon. After the completion of the reaction, the mixture was extracted with dichloromethane. The organic layers were separated, dried over magnesium sulfate, filtered, and concentrated. The residues were purified by silica gel column chromatography using *n*-hexan/dichloromethane as the eluent to give the target product *tris(4-(9-(4-(diphenylamino)phenyl)-9H-carbazol-2-yl)phenyl)amine* (**3c**). Yield: 30%; white solid; FT-IR (KBr pellet): ʋ_max_ 2359, 2337, 1509, 1490, 1278, 689 cm^−1^; ^1^H-NMR (CDCl_3_) δ 8.17 (dd, *J* = 8.7 Hz, 6H), 7.04–7.72 (m, 69H); ^13^C-NMR (CDCl_3_) δ 142.1, 141.5, 139.2, 129.6, 128.8, 128.0, 127.7, 127.1, 125.9, 125.0, 123.9, 123.6, 123.1, 122.5, 120.6, 120.3, 120.0, 119.6, 110.1, 109.9; Anal. Calcd for C_108_H_75_N_7_: C, 88.19; H, 5.14; N, 6.67. Found: C, 88.15; H, 5.93; N, 6.00.

#### 3.3. OLED Fabrication and Characterization

A glass substrate covered with indium tin oxide (ITO having a sheet resistance of 10 Ω/m^2^) was cleaned in ultrasonic baths containing acetone and 2-propanol, and rinsed in deionized water. The substrate was dried under a stream of nitrogen and subjected to a UV-ozone treatment. All organic and cathode metal layers were deposited by the vacuum deposition technique under a pressure of ~1 × 10^−7^ Torr. The deposition rate of the organic layers was about 0.5 Å/s. Then, LiF and Al were deposited in another vacuum deposition system without breaking the vacuum. Deposition rates of LiF and Al were 0.1 Å/s, and 5 Å/s, respectively. After deposition, the device was immediately encapsulated in ambient nitrogen. Current density-voltage (*J-V*) and luminance-voltage (*L-V*) characteristics of the device were measured by using a Keithley 2635A Source Meter Unit (SMU) and a Konica Minolta CS-100A. EL spectra and CIE color coordinates were obtained using a Konica Minolta CS-2000 spectroradiometer.

## 4. Conclusions

In summary, a series of novel HTMs based on 4-(*9H*-carbazol-9-yl)triphenylamine conjugated with different carbazole or triphenylamine derivatives have been synthesized and investigated. The resulting HTMs showed good morphological and thermal stabilities with high *T_g_* and *T_d_* values. Among these materials, HTM **3c** was found to be the best, exhibiting superior thermal properties and significantly enhanced device efficiencies as compared to the reference device using only NPB as HTM.
